# Nanoparticle formulation of mycophenolate mofetil achieves enhanced efficacy against hepatocellular carcinoma by targeting tumour‐associated fibroblast

**DOI:** 10.1111/jcmm.16434

**Published:** 2021-03-13

**Authors:** Zhentao Yang, Liang Zhang, Hai Zhu, Ke Zhou, Hangxiang Wang, Yuchen Wang, Rong Su, Danjing Guo, Lin Zhou, Xiao Xu, Penghong Song, Shusen Zheng, Haiyang Xie

**Affiliations:** ^1^ Division of Hepatobiliary and Pancreatic Surgery Department of Surgery First Afliated Hospital School of Medicine Zhejiang University Hangzhou China; ^2^ NHC Key Laboratory of Combined Multi‐organ Transplantation Hangzhou China; ^3^ Key Laboratory of the Diagnosis and Treatment of Organ Transplantation, Research Unit of Collaborative Diagnosis and Treatment for Hepatobiliary and Pancreatic Cancer Chinese Academy of Medical Sciences (2019RU019) Hangzhou China; ^4^ Key Laboratory of Organ Transplantation Hangzhou China

**Keywords:** cancer‐associated fibroblast, hepatocellular carcinoma, mycophenolate mofetil, nanoparticles

## Abstract

Hepatocellular carcinoma (HCC) is one of the most aggressive tumours with marked fibrosis. Mycophenolate mofetil (MMF) was well‐established to have antitumour and anti‐fibrotic properties. To overcome the poor bioavailability of MMF, this study constructed two MMF nanosystems, MMF‐LA@DSPE‐PEG and MMF‐LA@PEG‐PLA, by covalently conjugating linoleic acid (LA) to MMF and then loading the conjugate into polymer materials, PEG_5k_‐PLA_8k_ and DSPE‐ PEG_2k_, respectively. Hepatocellular carcinoma cell lines and C57BL/6 xenograft model were used to examine the anti‐HCC efficacy of nanoparticles (NPs), whereas NIH‐3T3 fibroblasts and highly‐fibrotic HCC models were used to explore the anti‐fibrotic efficacy. Administration of NPs dramatically inhibited the proliferation of HCC cells and fibroblasts in vitro. Animal experiments revealed that MMF‐LA@DSPE‐PEG achieved significantly higher anti‐HCC efficacy than free MMF and MMF‐LA@PEG‐PLA both in C57BL/6 HCC model and highly‐fibrotic HCC models. Immunohistochemistry further confirmed that MMF‐LA@DSPE‐PEG dramatically reduced cancer‐associated fibroblast (CAF) density in tumours, as the expression levels of alpha‐smooth muscle actin (α‐SMA), fibroblast activation protein (FAP) and collagen IV were significantly downregulated. In addition, we found the presence of CAF strongly correlated with increased HCC recurrence risk after liver transplantation. MMF‐LA@DSPE‐PEG might act as a rational therapeutic strategy in treating HCC and preventing post‐transplant HCC recurrence.

## 1 INTRODUCTION

Hepatocellular carcinoma (HCC) is the fourth leading cause of cancer‐related death worldwide, with a five‐year survival rate of only 18%.^1^ Despite advances in HCC administration, medical treatment still exhibits minimal benefit on patient survival.^2^ However, increasing evidence has documented that therapeutic agents targeting tumour microenvironment (TME) may provide a promising strategy for HCC treatment.^3^, ^4^, ^5^ Mycophenolate mofetil (MMF) is an extensively used immunosuppressive agent in liver transplantation (LT).^6^ In patients, MMF is quickly converted to an active metabolite, mycophenolic acid (MPA), which selectively inhibits inosine monophosphate dehydrogenase (IMPDH) and blocks the de novo biosynthesis of guanosine nucleotides.^7^ In addition to the anti‐rejection effect, increasing evidence has documented that MMF possesses antitumour potency and decreases the risk of developing malignancies following LT.^8^, ^9^, ^10^, ^11^ More importantly, recent studies have demonstrated that MPA exhibits a potent antifibrotic activity both in vitro and vivo.^12^ Treatment with MPA significantly inhibited hepatic stellate cells activation and reduced matrix accumulation in patients with chronic graft rejection.^13^, ^14^ It is well known that liver fibrosis or cirrhosis is closely related with HCC development as 80%‐90% HCC developed in livers with underlying fibrotic or cirrhotic background.^15^ Moreover, a group of activated fibroblasts called cancer‐associated fibroblast (CAF) have been proved to participate in the regulation of tumour microenvironment and greatly enhance the proliferation and metastasis of HCC, indicating that MMF may inhibit HCC growth via targeting these cells.^16^ In addition, LT has been widely accepted as an optimal treatment for HCC.^17^ Nevertheless, post‐transplant tumour recurrence greatly limits the efficacy of LT. Given the antitumour property of MMF, it may well be feasible to prevent HCC recurrence after LT by employing MMF. However, due to its poor water‐solubility, oral administration remains the only route for this agent in clinics. And it was reported that MMF exhibited variable antitumour effects in vivo, which probably due to its poor bioavailability.^18^ Hence, there is considerable motivation for the development of efficacious and safe approaches to deliver MMF in vivo, which could address the practical need of treating HCC and preventing post‐transplant HCC recurrence simultaneously.

Nanoparticle (NP)‐mediated drug delivery system offers great promise for cancer therapy by improving pharmacokinetic properties, facilitating accumulation within solid tumours and minimizing the off‐target and adverse effects.^19^, ^20^, ^21^ Numerous studies have been attempted to deliver therapeutic agents through NPs to achieve better anti‐CAF and anti‐HCC efficiency.^22^, ^23^ Amphiphilic polymer materials such as poly (ethylene glycol)‐block‐poly (D, L‐lactic acid) (PEG‐PLA) and 1, 2‐distearoyl‐sn‐glycero‐3‐phosphoethanolamine‐N‐poly (ethylene glycol) (DSPE‐ PEG) are widely used as biodegradable nano‐carriers to construct favourable delivery systems.^24^, ^25^ Upon amphiphilic assembly of mixed drugs and matrices, a core‐shell nanostructure can be obtained by packaging the hydrophobic drugs into the hydrophobic core whereas the hydrophilic components constitute the outer shell.^26^ Unfortunately, some therapeutic drugs have shown limited entrapment efficiency in these delivery materials due to their unfavourable physicochemical properties. In a preliminary experiment, we attempted to encapsulate the MMF agent into both mentioned matrices but failed to obtain stable nanoformulations.

In the present study, a structurally tailored MMF prodrug was constructed through chemical derivatization of the MMF compound with an unsaturated fatty acid, linoleic acid (LA). Compared with the parent MMF, the prodrug (MMF‐LA) showed enhanced lipophilicity which enabled its incorporation into amphiphilic copolymer materials to form stable NPs. For this purpose, PEG_5k_‐PLA_8k_ and DSPE‐PEG_2k_ were employed to establish the MMF‐LA@PEG‐PLA and MMF‐LA@DSPE‐PEG nanosystems, respectively. We then examined their antitumour activities in several HCC cell lines and a mouse model bearing HCC xenograft for evaluation of their clinical potential. Furthermore, in a preclinical CAF‐promoted HCC model, administration of nanotherapeutic, MMF‐LA@DSPE‐PEG, significantly decreased fibroblasts as well as tumour growth. To correlate the therapeutic potential of NPs with clinical data, we additionally examined the relationship between the CAF density accumulated in tumours and post‐transplant HCC recurrence risk by retrospectively collecting and analysing clinical data of HCC patients who had undergone LT in our hospital. These results highlighted the potential of traditional immunosuppressive agent MMF as a new anticancer therapeutic in treating HCC and preventing post‐LT HCC recurrence.

## 2 MATERIAL AND METHODS

### 2.1 Patient samples

Hepatocellular carcinoma samples were obtained from patients who had undergone deceased donor liver transplantation (DDLT) for HCC at the First Affiliated Hospital of Zhejiang University, China between January 2015 and October 2018. The study was performed in accordance with the Declaration of Helsinki and was approved by the Ethics Committee of the First Affiliated Hospital of Zhejiang University (Ethic code 2019‐1421). Tumour tissues were formalin fixed and then paraffin embedded for storage. The clinical characteristics of patients are listed in Table S1.

### 2.2 Cell culture

The human HCC cell lines Huh7, SUN 449, LM3, human hepatic stellate cell line LX2, mouse HCC cell line Hep1‐6 and mouse embryonic fibroblast cell line NIH‐3T3 were purchased from the Cell Bank of Chinese Academy of Sciences (Shanghai, China). All cell lines were cultured in DMEM (Thermo, USA) supplemented with 10% foetal bovine serum (FBS, Gibco, Australia) and maintained at 37°C in a humidified 5% CO_2_ condition.

### 2.3 Construction of MMF nano‐systems

To synthesize MMF‐LA (695 mg, 1 mmol), MMF (433 mg, 1 mmol) (MCE, USA) and LA (280 mg, 1 mmol) (sigma, USA) were reacted in 4 mL anhydrous N,N‐DiMethylformamide (DMF, J&Kseal, China) in the presence of 1‐(3‐Dimethylaminopropyl)‐3‐ethylcarbodiimide (EDC, J&Kseal, China) (155 mg, 1 mmol) and 4‐Dimethylaminopyridine (DMAP, TCI, China) (122 mg, 1 mmol). The reaction mixture was stirred at 43°C for 16 hours. Products were purified and obtained by flash column chromatography on silica gel. The detailed procedures were previously described.^27^ The MMF‐LA@DSPE‐PEG nanosystem was prepared by mixing MMF‐LA with DSPE‐PEG_2k_ at a ratio of 10:1 and then was injected into water under ultra‐sonication to yield working solution. To establish MMF‐LA@PEG‐PLA nano‐system, MMF‐LA was mixed with PEG_5k_‐PLA_8k_ at a ratio of 1:10 dissolved in 1 mL acetone. After completely mixing, the mixture was added into water dropwise and then the solution was further stirred at 45°C for 2 hours to remove the acetone. To compare the anti‐HCC and anti‐fibrosis efficiency between free MMF and the two MMF‐LA NPs at the same molar level of MMF, MMF‐LA and MMF were used at a mass ratio of 1.6/1 for further study, which was calculated according to their molecular weight (695.43/433.49 = 1.6043).

### 2.4 Characterization of NPs

The morphology of MMF‐LA NPs was characterized by transmission electron microscopy (TEM, Olympus, Japan). The size distribution and zeta potential of NPs were evaluated using dynamic light scattering (DLS, Malvner, UK).

### 2.5 Colony formation assay

See online supplementary Methods S1.

### 2.6 In vitro cytotoxicity assay

Cytotoxicity assay was performed using CCK‐8 (MCE, USA). Cells were seeded in 96‐well plates with a density of 2500‐3000 cells/well. After an overnight incubation, cells were treated with free MMF or MMF‐LA NPs in different concentrations. After 48 hours, CCK‐8 was carried out and the absorbance of each well at 450 nm was determined by spectrophotometer (Bio‐Rad, USA). IC50 was calculated according to the following method: *Y* = 100/(1 + [*X*/IC50]*^P^*). *Y* represents cell viability whereas *X* represents drug concentration, and the *P* value was .05.

### 2.7 Cell cycle analysis

Cells were seeded in 6‐well plates with a density of 1.5 × 10^4^ cells/well. After an overnight incubation, cells were treated with free MMF (2 µg/mL) or MMF‐LA NPs (at 2 µg/mL MMF‐equivalent dose) for 48 hours. Cells were harvested, washed with PBS and then fixed in 70% cold ethanol. One night later, cells were washed and incubated with propidium iodide (PI) for 30 minutes at room temperature. The relative proportion of cells in each cell cycle phase was analysed using flow cytometry.

### 2.8 Western blot analysis

NIH‐3T3 Cells were treated with free MMF (2 µg/mL) or MMF‐LA NPs (at 2 µg/mL MMF‐equivalent dose) for 48 hours. Cells were then collected and lysed using RIPA buffer (Thermo, USA). See online supplementary Methods S1.

### 2.9 Immunohistochemistry (IHC)

Tumours were harvested and fixed in 4% formaldehyde for one week prior the paraffin embedding. Immunohistochemistry was carried out as described previously.^27^ Primary antibodies used in this study were listed in the online supplementary Methods S1.

### 2.10 Immunofluorescence (IF)

NIH‐3T3 cells and LX2 cells were plated in confocal petri dishes. Cells were fixed by 4% PFA and then blocked with 5% BSA containing 0.3% Triton X‐100 for 1 hours. After blocking, cells were washed by PBS and incubated with primary antibody (rabbit anti‐α‐SMA (1:500) (CST, USA), mouse anti‐Tubulin (1:500) (CST, USA) and rabbit anti‐vimentin, (1:500) (CST, USA)) at 4°C overnight. After incubation, cells were washed and reacted with secondary antibody (1:200) (CST, USA) at room temperature for 1 hour. DAPI (Thermo, USA) was used to stain the nuclei.

### 2.11 Boyden chamber migration assay

See online supplementary Methods S1.

### 2.12 Animal experiments

Animals were purchased from Vital River Laboratory Animal Technology Co. Ltd (Beijing, China). All procedures involving animals were carried out in accordance with the ethical guidelines established by the Ethics Committee of the First Affiliated Hospital of Zhejiang University. To investigate the antitumour efficacy of MMF‐LA NPs in vivo, the HCC mouse model was constructed by subcutaneously injecting Hep1‐6 cells into the right flank of C57BL/6 mice. Mice were randomly divided into four groups as follows: Free‐MMF, MMF‐LA@PEG‐PLA, MMF‐LA@DSPE‐PEG and Control groups. Treatments were started when the tumour length reached approximately 3 mm. Mice were orally administrated with free MMF (20 mg/kg) or intravenously injected with MMF‐LA NPs (at 20 mg/kg MMF‐equivalent dose) every other day for four times. Tumour diameter (length and width) and bodyweights were measured and recorded every other day. Mice were killed when the tumour length reached 15 mm, and tumour samples were harvested for further study.

To confirm the tumour‐facilitating effect of CAF on HCC progression, a highly fibrotic HCC model (Model I) was established by co‐inoculating of Hep1‐6 HCC cells with NIH‐3T3 fibroblasts at a ratio of 2:1 into the right flank of nude mice. Before inoculation, NIH‐3T3 fibroblasts were activated by incubating with TGF‐β1 for 96 hours at a dose of 10 ng/mL.^28^ The anti‐CAF capacity of MMF‐LA NPs was then explored base on this model.

To confirm the anti‐CAF capacity of MMF‐LA NPs, another highly HCC model (Model II) was constructed by co‐inoculating LM3 HCC cells with LX2 cells, an activated human hepatic stellate cell line, at a ratio of 2:1 into the right flank of nude mice. Moreover, PDX models were established by using tumour samples collected from a patient who was diagnosed as HCC with liver cirrhosis.

### 2.13 CAF‐specific drug delivery

To examine the CAF targeting capability of MMF‐LA NPs, we specifically labelled MMF‐LA NPs by co‐assembling a Near‐infrared (NIR) dye, DiR, with MMF‐LA and DSPE‐PEG copolymer to generate MMF‐LA@DSPE‐PEG‐DiR nanosystem. The highly fibrotic HCC model (Model I) and non–highly fibrotic HCC model (without NIH‐3T3 cells) were constructed. When the tumour length reached 10 mm, free DiR and MMF‐LA@DSPE‐PEG‐DiR were injected respectively. After 24 hours, all mice were anesthetized and the fluorescence signal was detected using Clairvivo OPT (SHIMADZU Corporation, Japan).

### 2.14 Statistical analysis

Data were analysed using Prism 6 software and SPSS17.0 software. Shapiro‐Wilk test was used to determine the normality of variables. For normally distributed variables, Student's *t* test and one‐way ANOVA test followed with Tukey's HSD were applied for the comparison between two groups and among multiple groups, respectively. For variables non‐normally distributed, the comparison was performed using Mann‐Whitney U test. Tumour volume (*V*) was calculated as following: *V* = (*L* × *W* ^2^)/2, *L* represents length whereas *W* represents width. Chi‐square test was performed to determine the associations between CAF density and clinicopathological parameters. Hepatocellular carcinoma recurrence‐free survival (RFS) was analysed by Kaplan‐Meier analysis combined with log‐rank test. *P* value < .05 was considered statistically significant (*P < .05, **P < .01, ***P < .001, ****P < .0001).

## 3 RESULTS

### 3.1 Preparation and Characterization of MMF‐LA NPs

**FIGURE 1:**
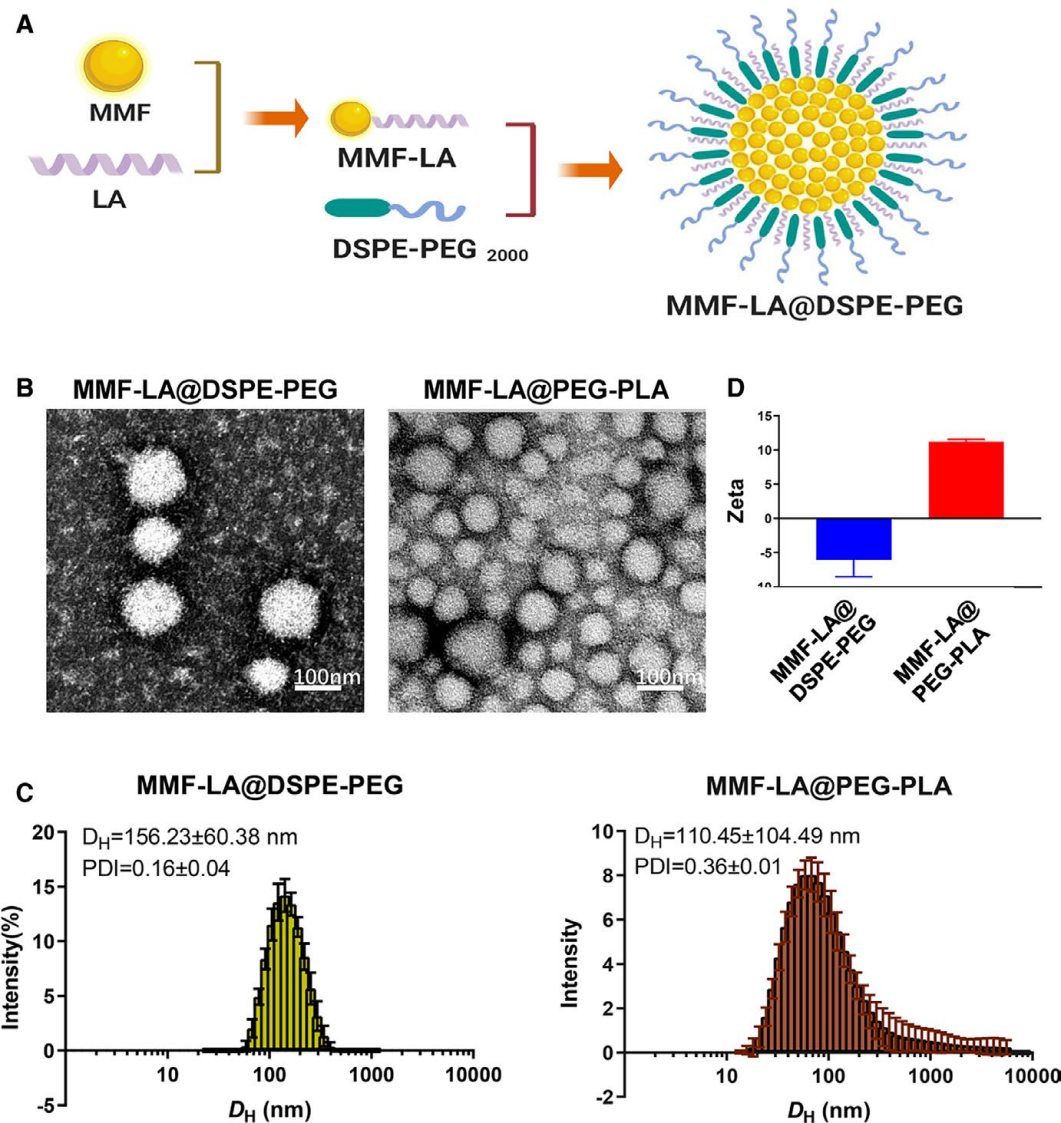
Construction and characteristics of MMF‐LA NPs. A, Schematic illustration of the construction of MMF‐LA@DSPE‐PEG nanosystem. MMF‐LA@DSPE‐PEG was constructed by covalently conjugating linoleic acid (LA) to mycophenolate mofetil (MMF) and then loading the conjugate into polymer material, DSPE‐PEG_2000_. B, The morphology of MMF‐LA@DSPE‐PEG and MMF‐LA@PEG‐PLA characterized by transmission electron microscopy (TEM). The scale bars: 100 nm. C and D, The diameters and zeta potentials of MMF‐LA@DSPE‐PEG and MMF‐LA@PEG‐PLA characterized by Dynamic light scattering (DLS)

To overcome the unfavourable hydrophobicity of MMF, we designed and synthesized a biodegradable prodrug of MMF by covalently conjugating LA to MMF via esterification. Compared with MMF, encapsulation of MMF‐LA within DSPE‐PEG_2k_ and PEG_5k_‐PLA_8k_ yielded stable NPs because of the physicochemical similarity between the prodrug and NP core. Schematic illustration of the construction of MMF‐LA@DSPE‐PEG nanosystem is shown in Figure 1A. The morphology and particle sizes of MMF‐LA@DSPE‐PEG and MMF‐LA@PEG‐PLA were characterized using TEM and DLS. As presented in Figure 1B, TEM‐based morphology studies indicated that both MMF‐LA@DSPE‐PEG and MMF‐LA@PEG‐PLA formed monodisperse and spherical nanostructures with small nanoscale sizes. Dynamic light scattering analysis revealed that the mean hydrodynamic diameters (*D*_H_) of MMF‐LA@DSPE‐PEG and MMF‐LA@PEG‐PLA were 156.23 ± 60.38 nm and 110.45 ± 104.49 nm, respectively. In contrast to the undesired polydispersity index (PDI > 0.2) of MMF‐LA@PEG‐PLA, the monomodal distribution and low PDI (<0.2) of MMF‐LA@DSPE‐PEG suggested the narrow size distribution of DSPE‐PEG encapsulated NPs (Figure 1C). In addition, the zeta potentials of MMF‐LA@DSPE‐PEG and MMF‐LA@PEG‐PLA were slightly negative and positive, respectively (Figure 1D).

### 3.2 In vitro cytotoxicity assays

**FIGURE 2:**
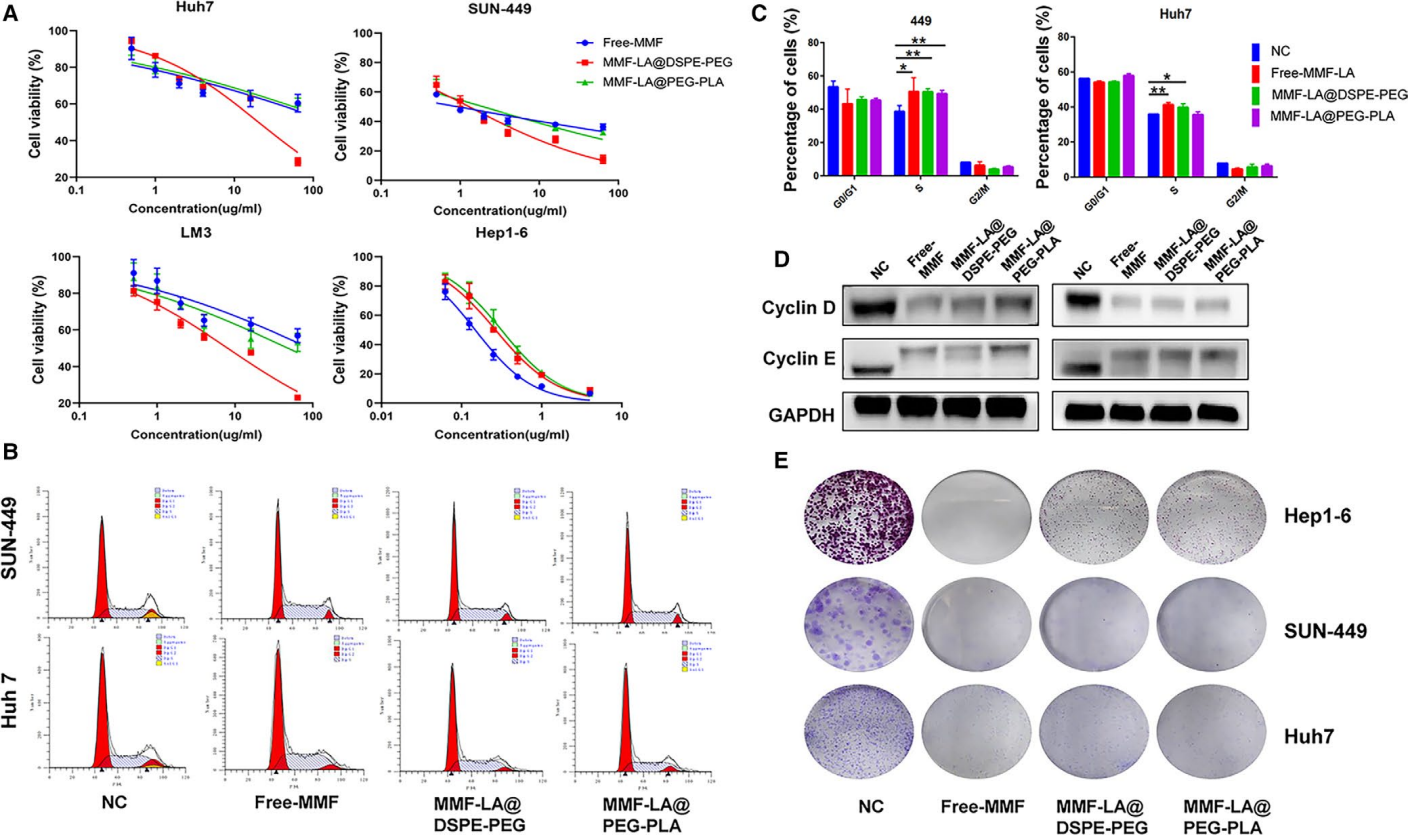
Anti‐HCC efficiency of MMF‐LA NPs in vitro. A, Cell viabilities of Huh7 cells, SUN‐449 cells, LM3 cells and Hep1‐6 cells treated with free MMF, MMF‐LA@DSPE‐PEG and MMF‐LA@PEG‐PLA for 48 h. B, Cell cycles determined by flow cytometry. Cells were treated with free MMF (2 µg/mL) or MMF‐LA NPs (at 2 µg/mL MMF‐equivalent dose) for 48 h. C, Quantitative analysis of the results in panel B. Data are shown as the mean ± SD (n = 3), *P < .05, **P < .01. D, Expression levels of cyclin D and cyclin E determined using Western blot. E, Colony Formation of Hep1‐6 cells, SUN‐449 cells and Huh7 cells. Hep1‐6 cells, SUN‐449 cells and Huh7 cells were treated with drugs at 0.5, 2 and 4 µg/mL (MMF‐equivalent concentration), respectively, for 96 h. All the results are representative of at least three independent experiments

We next examined the cytotoxicity of MMF‐LA@DSPE‐PEG, MMF‐LA@PEG‐PLA and free MMF against a panel of HCC cell lines (ie human HCC cell lines Huh7, SUN 449, LM3 and mouse HCC cell line Hep1‐6) by evaluating the cell viability via CCK‐8 assay. As shown in Figure 2A, MMF‐LA NPs displayed comparable or even enhanced cytotoxicity against human HCC cell lines than free MMF in vitro. The half‐maximal inhibitory concentration (IC50) for MMF‐LA@DSPE‐PEG, MMF‐LA@PEG‐PLA and free MMF was 20.40, 210.60 and 175.90 µg/mL, respectively, in Huh7 cell line, 1.29, 1.94 and 0.91 µg/mL, respectively, in SUN‐449 cell line, 8.09, 48.44 and 92.55 µg/mL, respectively, in LM3 cell line and 0.26, 0.31 and 0.15 µg/mL, respectively, in Hep1‐6 cells. Inspiringly, high dose of MMF‐LA@DSPE‐PEG achieved dramatically higher anti‐HCC efficacy than MMF‐LA@PEG‐PLA and free MMF in all human HCC cell lines, indicating the enhanced cytotoxicity of MMF by encapsulating with DSPE‐PEG. As a highly selective inhibitor of inosine monophosphate dehydrogenase (IMPDH), MPA effectively disrupt the de novo biosynthesis of guanosine nucleotides. Given this, we investigated the impact of MMF on cell cycle progression of HCC cells using flow cytometric analyses. As shown in Figure 2B,C, both MMF‐loaded nanotherapeutics and free MMF significantly increased the cell proportion of S phase in SUN‐449 and Huh7 cells, indicating the blockage of DNA replication in these cells. We also examined the expression status of several key cyclins that regulating cell cycle progression. Western blot revealed that the expression of cyclin D and cyclin E were remarkably decreased after treatments with free MMF and nanotherapeutics (Figure 2D). Colony formation assay further indicated that MMF‐LA@DSPE‐PEG, MMF‐LA@PEG‐PLA and free MMF dramatically inhibited the colony formation of Hep1‐6, SUN449 and Huh7 cells (Figure 2E). Together, these results demonstrated that both MMF‐LA NPs showed comparable capacity in inducing cytotoxicity and preventing cell proliferation with free MMF against HCC cell lines, suggesting that no obvious reduction of toxicity was observed after constructing the MMF into nanoformulations.

### 3.3 MMF‐LA@DSPE‐PEG inhibited HCC growth in vivo

**FIGURE 3:**
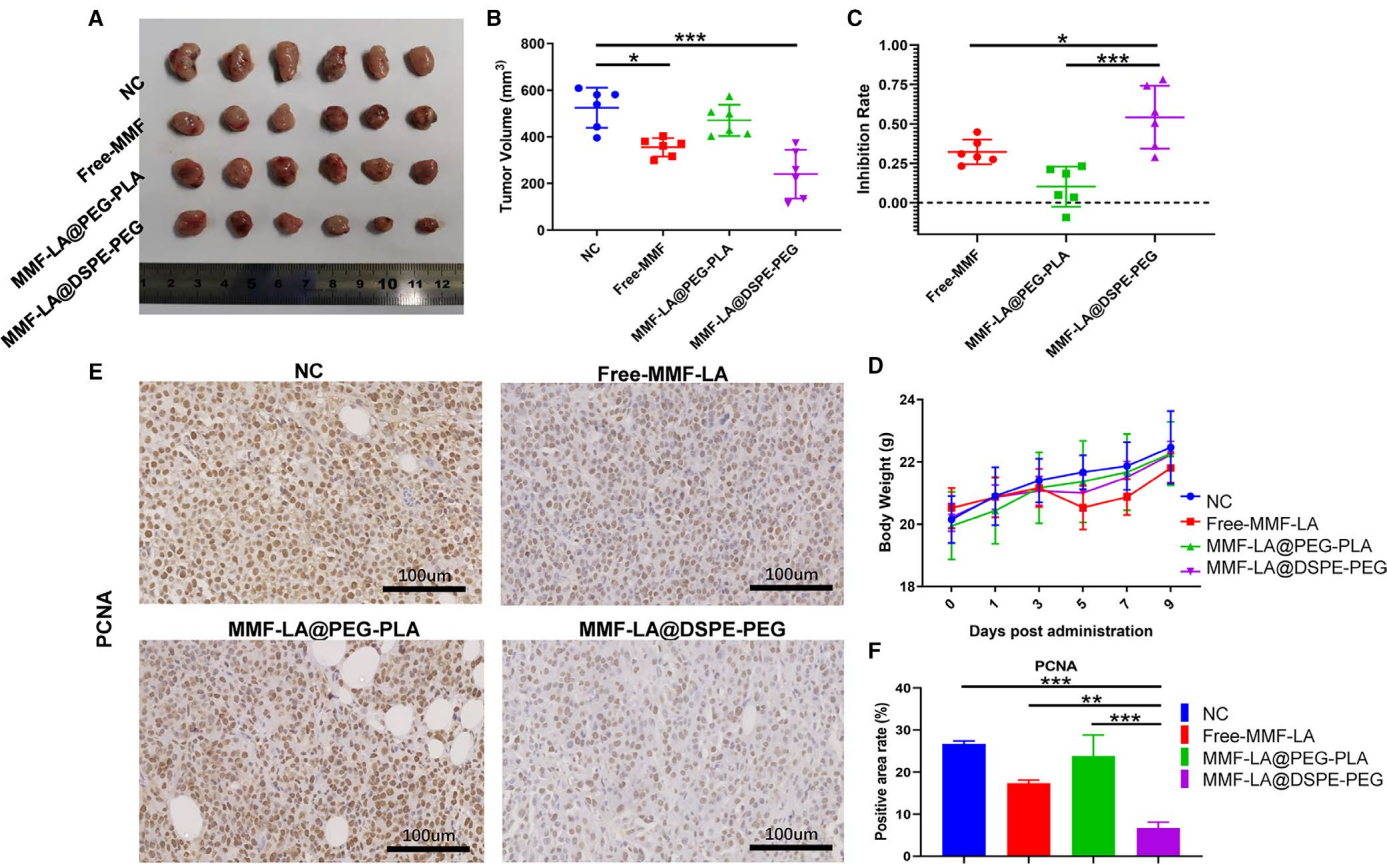
Anti‐HCC efficiency of MMF‐LA NPs in vivo. A, Images of Hep1‐6 tumours after treatment with drugs at 20 mg/kg (MMF‐equivalent concentration), (n = 6). B, Tumour volumes of different groups (n = 6), *P < .05, ***P < .001. C, Tumour inhibition rates (*R*) of different treatments. $R=(\bar{v}nc‐v/\bar{v}nc)$, $\bar{v}nc$ represents the mean tumour volume of NC group, *v* represents the tumour volume of free MMF, MMF‐LA@DSPE‐PEG or MMF‐LA@PEG‐PLA groups, (n = 6), *P < .05, ***P < .001. D, Bodyweights (mean ± SD, n = 6) of mice in different groups. E, Expression levels of PCNA in different groups determined by Immunohistochemistry. The scale bars: 100 µm. F, Quantitative analysis of PCNA expression status in panel E (Image J software), data are shown as the mean ± SD, (n = 3), **P < .01, ***P < .001

As a commonly used immunosuppressive agent, MMF is known to effectively inhibit the proliferation of lymphocytes, which may affect individual antitumour immune response. Thus, the potential regulation of MMF on immune system should be taken into account when interpreting the antitumour activity of MMF. Given this, we explored the anti‐HCC efficacy of MMF‐LA NPs in vivo using C57BL/6 mouse model subcutaneously bearing Hep1‐6 xenografts, the immune system of which is preserved. Following systemic administration of MMF‐LA@DSPE‐PEG, MMF‐LA@PEG‐PLA and free MMF, the tumour volumes and inhibition rates of each group were calculated and recorded, as shown in Figure 3A‐C. Treatments with MMF‐LA@DSPE‐PEG and free MMF remarkably retarded tumour growth whereas treatment with MMF‐LA@PEG‐PLA showed negligible efficacy. Notably, MMF‐LA@DSPE‐PEG achieved better anti‐HCC efficacy than free MMF in tumour inhibition rate, which might be contributed to the long‐term blood circulation and passive tumour‐targeting capacity via enhanced permeability and retention (EPR) effect. In addition, treatment with free MMF caused a large drop of bodyweight at day 5, whereas the stable bodyweight of the mice receiving MMF‐LA NPs indicated that nanoformulations alleviated the in vivo toxicity induced by MMF (Figure 3D). We further examined the proliferation activity of tumours by examining the expression status of PCNA using IHC. As shown in Figure 3E,F, the PCNA expression was dramatically suppressed in MMF‐LA@DSPE‐PEG treated group, confirming the anti‐HCC capacity of this nanosystem.

### 3.4 In vitro anti‐fibrotic assays

**FIGURE 4:**
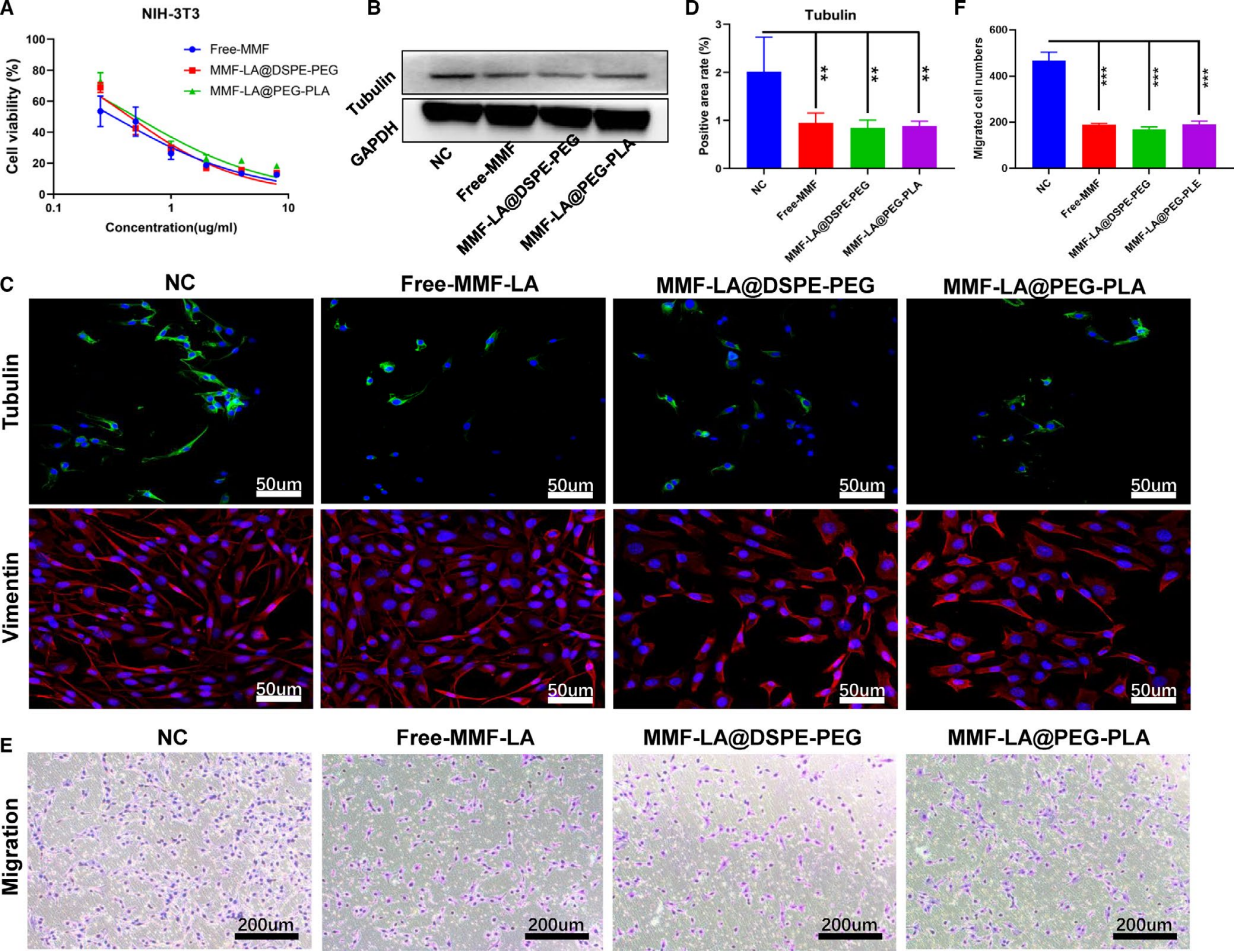
Anti‐fibrotic efficiency of MMF‐LA NPs in vitro. A, Cell viability of NIH‐3T3 cells treated with free MMF, MMF‐LA@DSPE‐PEG and MMF‐LA@PEG‐PLA for 48 h. B, Expression levels of tubulin determined by Western blot. C, Expression levels of tubulin and cell morphology of fibroblasts determined by Immunofluorescence. Green: tubulin; red: vimentin, blue: nuclei. The scale bars: 50 µm. D, Quantitative analysis of tubulin expression status in panel C (Image J software), data are shown as the mean ± SD, (n = 3), **P < .01. E, NIH‐3T3 cells were seeded in the top chamber of transwell with serum‐free medium and treated with free MMF, MMF‐LA@DSPE‐PEG and MMF‐LA@PEG‐PLA. After 24 h, migrated cells were fixed, stained and photographed. The scale bars: 200 µm (F) Quantitative analysis of the migrated cell number, data are shown as the mean ± SD, (n = 3), ***P < .001

Considering the prominent role of fibrosis in HCC tumorigenesis, we explored the anti‐fibrotic activity of MMF‐LA NPs using NIH‐3T3 fibroblasts. As shown in Figure 4A, MMF‐LA@DSPE‐PEG, MMF‐LA@PEG‐PLA and free MMF showed comparable cytotoxicity, and the IC50 was 0.45, 0.49 and 0.32 µg/mL, respectively. Western blot revealed that expression level of tubulin was remarkably suppressed after drug administration (Figure 4B). Then, IF analysis was performed to verify the expression status of tubulin, and we also investigated the potential impact of MMF on cellular morphology by examining vimentin. As shown in Figure 4C,D, IF confirmed the reduced expression level of tubulin, and more importantly, the classic spindle‐shape morphology of fibroblasts was dramatically damaged after incubation with drugs, especially the MMF‐LA@DSPE‐PEG group. Furthermore, transwell migration assay showed that MMF‐LA@DSPE‐PEG, MMF‐LA@PEG‐PLA and free MMF significantly reduced migratory activity of NIH‐3T3 cells (Figure 4E,F).

### 3.5 CAF promote HCC growth and increase HCC recurrence risk after liver transplantation

**FIGURE 5:**
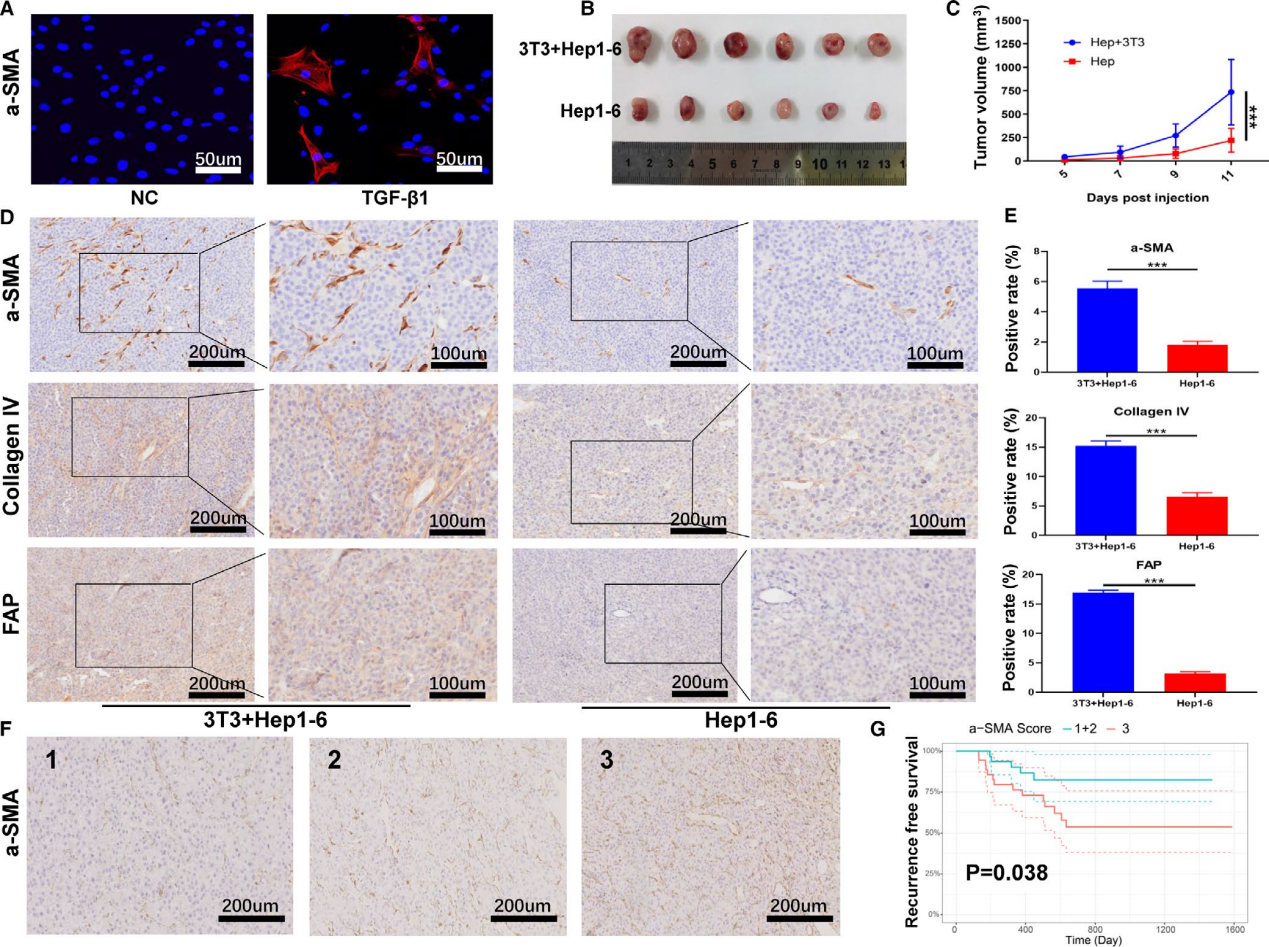
CAF significantly enhanced HCC growth in vivo. A, Expression levels of α‐SMA determined by Immunofluorescence. NIH‐3T3 fibroblasts were co‐cultured with TGF‐β1 or cultured alone for 96 h. B, Tumour images of different groups. Hep1‐6 cells were injected with activated fibroblasts or injected alone into the nude mice, (n = 6). C, Tumour growth curves of different groups, ***P < .001. D, Expression levels of α‐SMA, FAP and collagen IV determined by Immunohistochemistry. The scale bars: 50, 100 or 200 µm. E, Quantitative analysis of panel D (Image J software), data are shown as the mean ± SD, (n = 3), ***P < .001. F, Representative images showing low α‐SMA expression (CAF density = 1), median α‐SMA expression (CAF density = 2) and high α‐SMA expression (CAF density = 3) in HCC samples obtained from HCC patients underwent liver transplantation. G, Kaplan‐Meier analysis of patients with moderate CAF density (group 1 + 2) and high CAF density (group 3)

To confirm the HCC‐promoting efficacy of CAF, we constructed a highly fibrotic HCC model by subcutaneously injecting Hep1‐6 HCC cells with NIH‐3T3 fibroblasts into Balb/c nude mice. To acquire similar function of CAF, NIH‐3T3 fibroblasts were pre‐activated by incubating with TGF‐β1 for 96 hours and were proved to express alpha‐smooth muscle actin (α‐SMA), a widely accepted marker of activated fibroblasts and CAF before injection (Figure 5A). As expected, HCC cells injected with activated fibroblasts exhibited greater tumorigenesis potential compared with the HCC cells injected alone (Figure 5B,C). Immunohistochemistry analysis further confirmed the increased expression levels of α‐SMA, fibroblast activated protein (FAP) and collagen IV within tumours (Figure 5D,E). We then explored the relationship between CAF and post‐transplant HCC recurrence. Clinical data of 68 HCC patients who received LT at our hospital were retrospectively collected and the CAF density in HCC samples obtained from these patients was determined using IHC for α‐SMA. According to the proportion of α‐SMA‐positive cells accumulated in tumours, the CAF density was scored as 1, 2 or 3, and the representative images are shown in Figure 5F. Kaplan‐Meier analysis was performed and the result showed that patients with moderate CAF density (score 1 + 2) had significantly superior RFS rates than those abundant with CAF (score 3) (Figure 5G). The 1‐ and 3‐year RFS rates were 90.2% and 82.4%, respectively, in score 1 + 2 group, and the corresponding values were 76.3% and 53.6%, respectively, in score 3 group. Moreover, the associations between CAF density and clinical parameters were investigated. As shown in Table S2, high CAF density was associated with significantly increased proportion of microvascular invasion (13 vs 3, *P* = .009). And patients with low CAF density tended to have better differentiated tumours, though the difference was not statistically significant (4 vs 0, *P* = .089).

### 3.6 MF‐LA@DSPE‐PEG inhibited HCC growth by suppressing CAF

**FIGURE 6:**
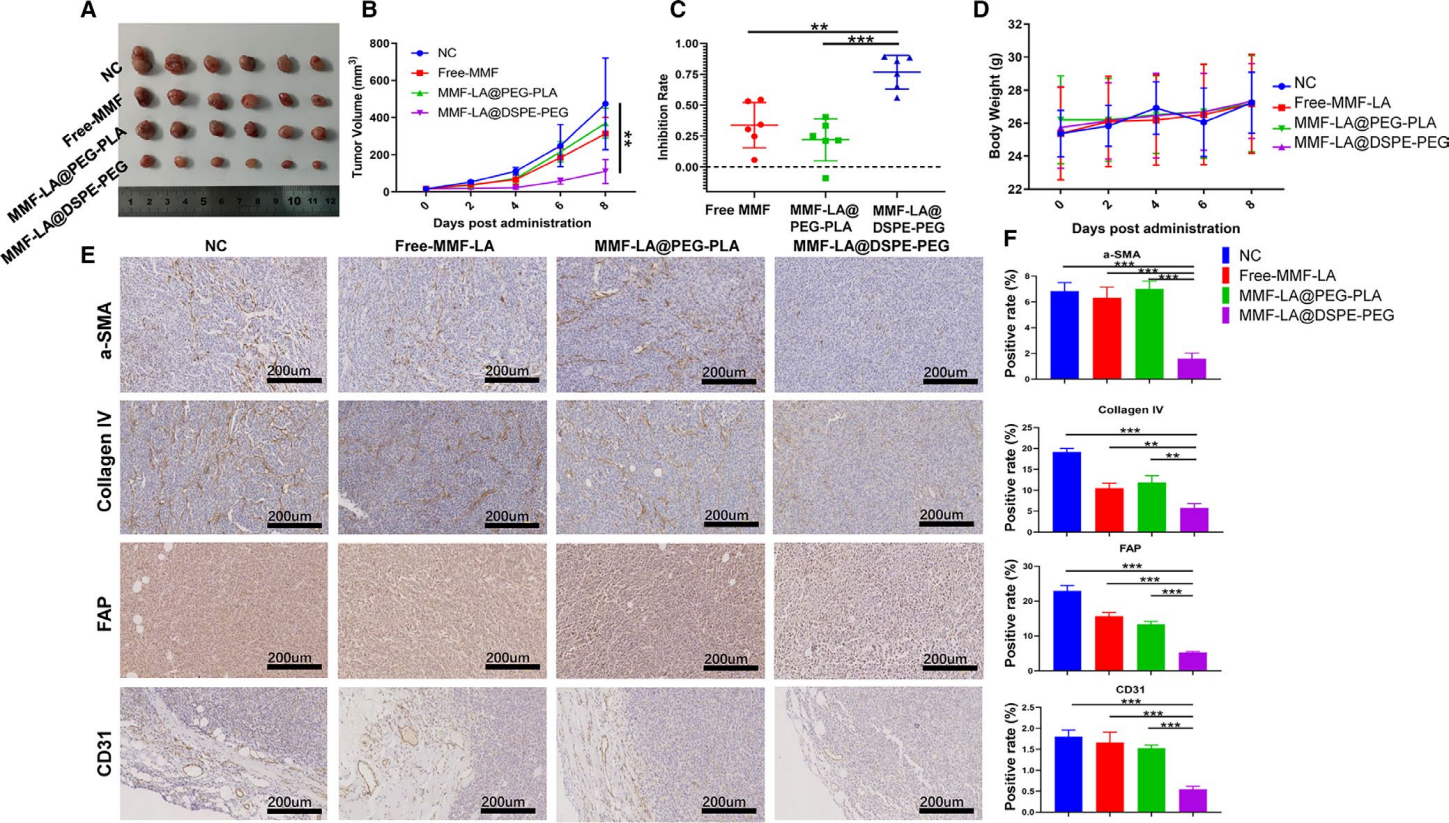
MMF‐LA@DSPE‐PEG inhibited HCC growth by depleting CAF. Mice were orally administrated with free MMF (20 mg/kg) or intravenously injected with MMF‐LA NPs (at 20 mg/kg MMF‐equivalent dose) every other day for four times. A, Tumour images of different groups, (n = 6). B, Tumour growth curves of different groups, **P < .01. C, Tumour inhibition rates of different treatments. (n = 6), **P < .01, ***P < .001. D, Bodyweights (mean ± SD, n = 6) of mice in different groups. E, Expression levels of α‐SMA, FAP, collagen IV and CD31 determined by Immunohistochemistry. The scale bars: 200 µm. F, Quantitative analysis of panel E (Image J software), data are shown as the mean ± SD, (n = 3), **P < .01, ***P < .001

We next investigated the anti‐HCC and anti‐CAF activity of nano‐MMF based on the established highly‐fibrotic HCC model (Model I). As shown in Figure 6A‐C, MMF‐LA@DSPE‐PEG dramatically inhibited HCC growth whereas free MMF and MMF‐LA@PEG‐PLA exhibited negligible anti‐HCC activity. In this experiment, the bodyweight of mice in all groups remained stable, indicating the low systemic toxicity of these treatments (Figure 6D). Immunohistochemistry demonstrated that proportion of CAF accumulated in tumours was remarkably downregulated following the treatment of MMF‐LA@DSPE‐PEG, as the expression levels of α‐SMA, FAP and collagen IV were significantly decreased (Figure 6E,F). In addition, MMF‐LA@DSPE‐PEG significantly reduced the expression levels of CD 31, underlying the decreased vascular density in tumours.

To further verify the anti‐CAF efficiency of nano‐MMF, we constructed another highly fibrotic HCC model by co‐inoculating LM3 HCC cells with LX2 cells, an activated human hepatic stellate cell line, at a ratio of 2:1 into the right flank of nude mice (Model II). Immunofluorescence confirmed that LX2 cells high expressed a‐SMA and Tubulin (Figure S1a). CCK8 assay and Transwell migration assay further revealed the cytotoxicity and migration‐inhibitive effect of MMF‐LA NPs and Free‐MMF on these cells (Figure S1b‐d). The anti‐HCC and anti‐CAF activity of nano‐MMF was then explored on this model. As shown in Figure S2a‐c, both free MMF and MM‐LA NPs significantly inhibited tumour growth, whereas MMF‐LA@DSPE‐PEG showed much higher efficiency against tumours when compared with free MMF and MMF‐LA@PEG‐PLA. Immunohistochemistry further confirmed that MMF‐LA@DSPE‐PEG dramatically reduced CAF density in tumours (Figure S2e,f). And the bodyweight of mice in all groups remained stable (Figure S2d). We also established PDX models by using tumour samples obtained from a patient diagnosed as HCC with liver cirrhosis, and the similar results were observed (Figure S3a‐f).

**FIGURE 7:**
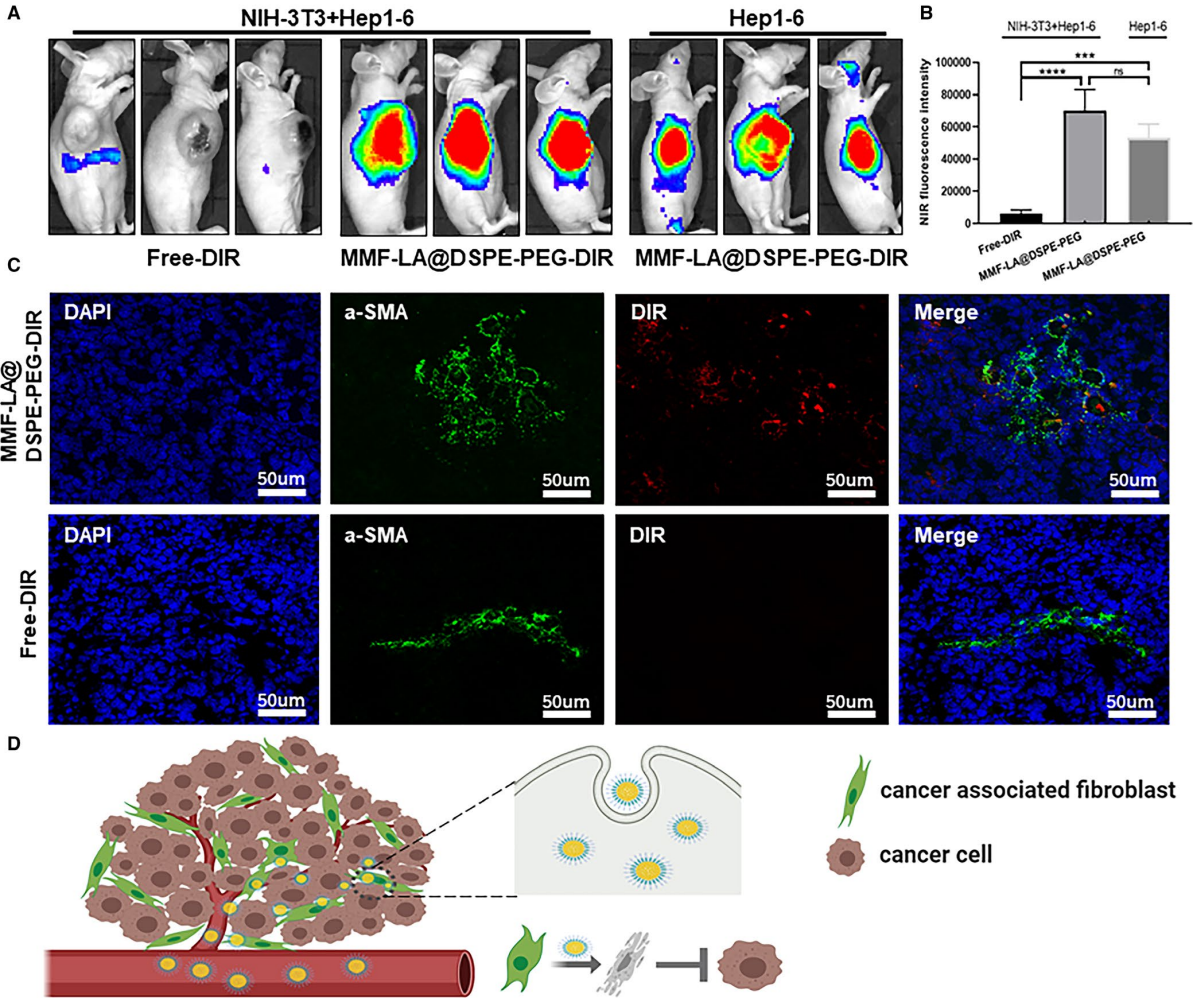
CAF‐targeting capacity of MMF‐LA@DSPE‐PEG. A, Accumulation of Free‐DIR and MMF‐LA@DSPE‐PEG‐DIR within tumours. B, Quantitative analysis of fluorescence intensity, n = 3, ***P < .001. C, Colocalization of MMF‐LA@DSPE‐PEG‐DIR and CAF. Green: α‐SMA; red: MMF‐LA@DSPE‐PEG‐DIR, blue: nuclei. The scale bars: 50 µm. D, Schematic illustration of the working mechanism of MMF‐LA@DSPE‐PEG. Cancer‐associated fibroblast (CAF) greatly enhance tumour growth. The established MMF‐LA@DSPE‐PEG nanoparticles effectively target CAF and then are internalized by these cells. As a consequence, CAF are dramatically suppressed and CAF‐related tumour growth is inhibited

Given the above results, we finally explored the cellular uptake of MMF‐LA@DSPE‐PEG by CAF within tumours to further confirm the high CAF‐targeting capacity of MMF‐LA@DSPE‐PEG. A Near‐infrared (NIR) dye, DiR, was used to specifically label the NPs by co‐assembling with MMF‐LA and DSPE‐PEG copolymer to generate MMF‐LA@DSPE‐PEG‐DiR nanosystem. As expected, strong fluorescence signals were observed in tumours treated with MMF‐LA@DSPE‐PEG‐DiR, whereas Free‐DiR exhibited negligible tumour accumulation (Figure 7A,B). Furthermore, IF showed that the majority of MMF‐LA@DSPE‐PEG‐DiR accumulated in the α‐SMA positive area, confirming the high CAF‐targeting capacity of MMF‐LA@DSPE‐PEG (Figure 7C). Schematic illustration of the working mechanism of MMF‐LA@DSPE‐PEG is shown in Figure 7D.

## 4 DISCUSSION

Mycophenolate mofetil can be rapidly hydrolysed into MPA by esterase in circulation.^29^ The generated MPA selectively inhibits IMPDH which is absolutely required in the de novo pathway of purine generation, inducing multiple cell effects.^30^ Among these effects, the antitumour property of MPA has aroused the interest of scientists since the late 1960s.^31^ However, previous studies have reported that MMF only marginally inhibited tumour progression in vivo due to its poor drug availability.^18^ In this study, we established a combinatorial strategy of facilely constructing MMF with LA, one of the polyunsaturated fatty acids (PUFAs), to obtain a biodegradable prodrug MMF‐LA, and sequentially co‐assembled with amphiphilic polymer materials PEG_5k_‐PLA_8k_ and DSPE‐PEG_2000_, respectively. ‘PUFAylation’ endowed the MMF‐based nanotherapeutics improved stability, prolonged blood circulation and reduced toxicity. Previous results confirmed that MMF‐LA@DSPE‐PEG and MMF‐LA@PEG‐PLA significantly inhibited HCC proliferation, arrested cell cycles in the S‐phase and inhibited the colony formation. The superior anti‐HCC activity of MMF‐LA@DSPE‐PEG was also verified in C57BL/6 mouse model subcutaneously bearing Hep1‐6 xenografts, as tumour growth was significantly retarded after the treatments. However, MMF‐LA@PEG‐PLA showed negligible restraint against Hep1‐6 tumour growth which might attributed to the wide distribution of particle sizes resulting in decreased tumour accumulation.

As a dominant component of TME, CAF has been proved to play a prominent functional role in tumour progression.^32^ In the context of HCC, the presence of activated hepatic stellate cells (HSC) which was considered as the major origin of CAF in liver was strongly associated with the poor prognosis of patients received curative resection.^33^ CAF affects HCC tumorigenesis and metastasis through various of methods, such as secreting tumour‐promoting cytokines, regulating extracellular matrix (ECM) stiffness, promoting epithelial to mesenchymal transition (EMT) and reducing immune surveillance.^16^ For instance, CAF‐secreted chemokines like CCL7 and CXCL16 strongly induced the activation of TGF‐β pathway in HCC, which significantly accelerated tumour metastasis.^34^ In addition, it was demonstrated that CAF promoted the tumour‐initiating cell plasticity of HCC through the activation of HGF/c‐Met cascade thus enhanced HCC tolerance to chemotherapeutic agents.^35^ To confirm the high HCC‐promoting efficacy of CAF, the present study established a highly‐fibrotic HCC model by co‐inoculating of Hep1‐6 HCC cells with NIH‐3T3 fibroblasts at a ratio of 2:1 into nude mice. Before co‐inoculation, we noticed that NIH‐3T3 fibroblasts did not normally express α‐SMA, a widely accepted marker of activated fibroblast and CAF, indicating that NIH‐3T3 fibroblasts remained inactive status. To better mimic the CAF function, we co‐cultured NIH‐3T3 fibroblasts with TGF‐β1 for 96 hours before injection as previous studies had reported that TGF‐β1 was a dominant profibrotic cytokine in the activation of fibroblasts and the progression of fibrosis.^36^ The expression status of α‐SMA was then verified by IF. As expected, NIH‐3T3 fibroblasts started to express α‐SMA after stimulation of TGF‐β1, underlying these cells were successfully activated. Based on the highly fibrotic HCC model, we confirmed that CAF dramatically enhanced HCC growth as HCC cells injected with activated fibroblasts showed greater tumorigenesis potential compared with the HCC cells injected alone.

Given the high HCC‐promoting efficacy of CAF, we suggested that CAF might influence the LT outcome by increasing HCC recurrence risk. To verify this hypothesis, clinical data of 68 HCC patients who had undergone LT at our hospital were retrospectively collected and analysed. As expected, the results revealed that patients with low CAF density had significantly better RFS rates. Moreover, high CAF density was associated with significantly increased proportion of microvascular invasion, which was consistent with the previous studies that CAF greatly promoted HCC migration and invasion.^16^, ^37^ Our results suggested that CAF might increase HCC recurrence risk after LT by facilitating the incidence of microvascular invasion, an independent risk factor for recurrence.^17^ Therefore, depleting CAF might be a rational strategy to suppress HCC growth and metastasis, and decrease post‐LT HCC recurrence risk.

Interestingly, accumulating evidence suggests that MMF possesses remarkable anti‐fibrotic property. For instance, co‐culture human mesangial cells with MPA not only inhibited cell proliferation but also decreased the fibronectin and Collagen I deposition induced by profibrotic cytokines.^38^ Using fibroblasts isolated from rejected rat cardiac allografts, Johnsson et al found that MPA might be used to delay allograft fibrosis as it almost totally inhibited fibroblasts proliferation.^39^ Given these, we explored the anti‐fibrotic efficacy of nano‐MMF using NIH‐3T3 fibroblasts and the established highly fibrotic HCC models. The results revealed that all forms of MMF dramatically inhibited fibroblasts proliferation and tubulin expression in vitro. Moreover, MMF‐LA@DSPE‐PEG significantly suppressed tumour growth and dramatically reduced CAF density in tumours when compared with free MMF and MMF‐LA@PEG‐PLA. In addition, MMF‐LA@DSPE‐PEG significantly inhibited the process of angiogenesis which was proved to promote tumour metastasis.^40^ We next investigated the CAF‐targeting capacity of MMF‐LA@DSPE‐PEG to further confirm its anti‐fibrotic efficacy. As expected, MMF‐LA@DSPE‐PEG majorly accumulated in the α‐SMA positive area, underlining that most of MMF‐LA@DSPE‐PEG NPs were up‐taken by CAF.

Despite the development of HCC treatment in the last decades, only one chemotherapeutic agent, sorafenib, was demonstrated to improve patient survival.^41^ Therefore, exploring novel targets beside HCC cells may provide feasible strategies in treating HCC. Given the pivotal role of microenvironment in HCC development, it is a strong rationale to modulate the crosstalk between HCC cells and stroma cells. Interestingly, the established MMF‐LA@DSPE‐PEG NPs not only inhibited HCC cells directly but also modulated HCC‐promoting microenvironment by suppressing CAF and decreasing vascular density. In addition, MMF is widely used as a therapeutic agent in various of immune‐disordered diseases.^42^ And it is well known that HCC is closely associated with immune disregulation especially the chronic inflammation, suggesting the possible application of MMF in treating HCC. In this study, we also demonstrated that high CAF density was strongly associated with increased proportions of microvascular invasion and patients with high CAF density had significantly inferior RFS rates. Collectively, we believe that MMF‐LA@DSPE‐PEG could achieve great anti‐HCC efficacy by effectively depleting CAF and also be utilized as an ideal strategy to prevent HCC recurrence after LT.

In conclusion, we described a simple approach for constructing a systemically injectable MMF‐based nanoplatform by covalently conjugating the MMF with LA followed by co‐assembling with amphiphilic copolymer. We demonstrated that the optimized nanotherapeutic MMF‐LA@DSPE‐PEG NPs exhibited enhanced cytotoxicity against a panel of human HCC cell lines and achieved improved anti‐HCC efficacy in vivo by increasing tumour accumulation, killing HCC cells, suppressing CAF and inhibiting tumour angiogenesis, as compared to free MMF. We envision that the MMF‐LA@DSPE‐PEG NPs could find a practical application in treating HCC and preventing post‐transplant HCC recurrence.

## CONFLICT OF INTEREST

The authors confirm that there are no conflicts of interest.

## AUTHOR CONTRIBUTIONS

**Zhentao Yang:** Data curation (equal); Formal analysis (equal); Investigation (lead); Methodology (equal); Writing‐original draft (equal). **Liang Zhang:** Formal analysis (equal); Investigation (lead); Methodology (equal); Writing‐original draft (equal). **Hai Zhu:** Data curation (equal); Investigation (supporting); Methodology (equal); Software (equal). **Ke Zhou:** Formal analysis (equal); Investigation (supporting); Methodology (supporting). **Hangxiang Wang:** Conceptualization (supporting); Investigation (supporting); Methodology (supporting). **Yuchen Wang:** Investigation (supporting); Methodology (supporting); Writing‐original draft (supporting). **Rong Su:** Investigation (supporting); Methodology (supporting). **Danjing Guo:** Investigation (supporting); Methodology (supporting). **Lin Zhou:** Funding acquisition (supporting); Project administration (supporting); Resources (supporting); Supervision (supporting). **Xiao Xu:** Funding acquisition (supporting); Project administration (supporting); Resources (supporting); Supervision (supporting). **Penghong Song:** Project administration (supporting); Supervision (supporting); Visualization (supporting). **shusen zheng:** Conceptualization (lead); Funding acquisition (lead); Project administration (lead); Resources (lead); Supervision (lead); Writing‐review and editing (lead). **Haiyang Xie:** Conceptualization (lead); Funding acquisition (lead); Project administration (lead); Resources (lead); Supervision (lead); Writing‐review and editing (lead).

## Supporting information

Figure S1

Figure S2

Figure S3

Table S1

Table S2

Method S1

FigCaption

## Data Availability

All data included in this study are available upon reasonable request by contact with the corresponding authors.
