# Supplementary figures and images for: Nanoparticle formulation of mycophenolate mofetil achieves enhanced efficacy against hepatocellular carcinoma by targeting tumour‐associated fibroblast
Source: J Cell Mol Med. 2021 Mar 13;25(7):3511–23. doi: 10.1111/jcmm.16434 (PMC8034467; doi:10.1111/jcmm.16434)

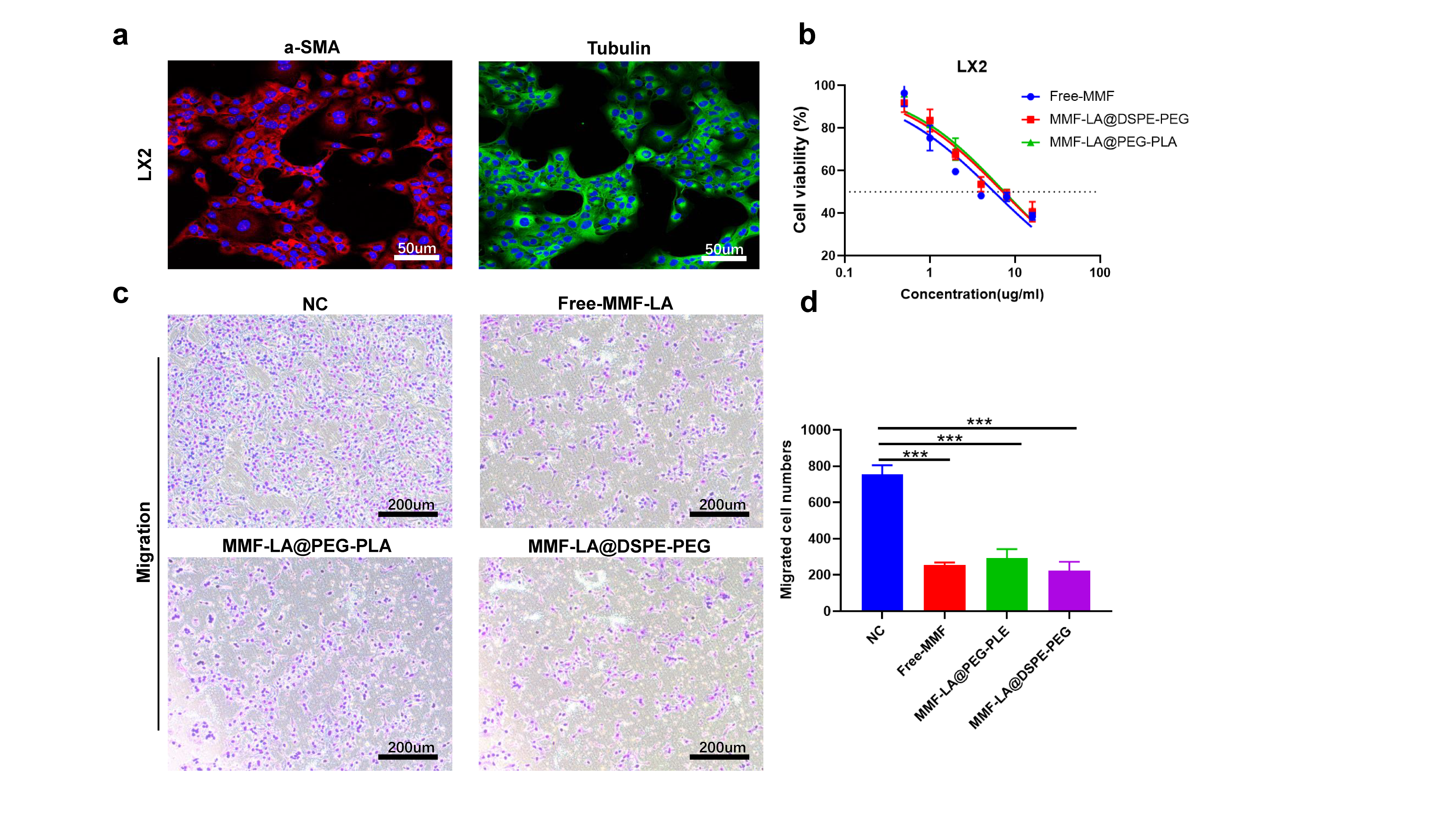

Supplement: Supplementary file 1 — Figure S1 [file JCMM-25-3511-s005.tif]

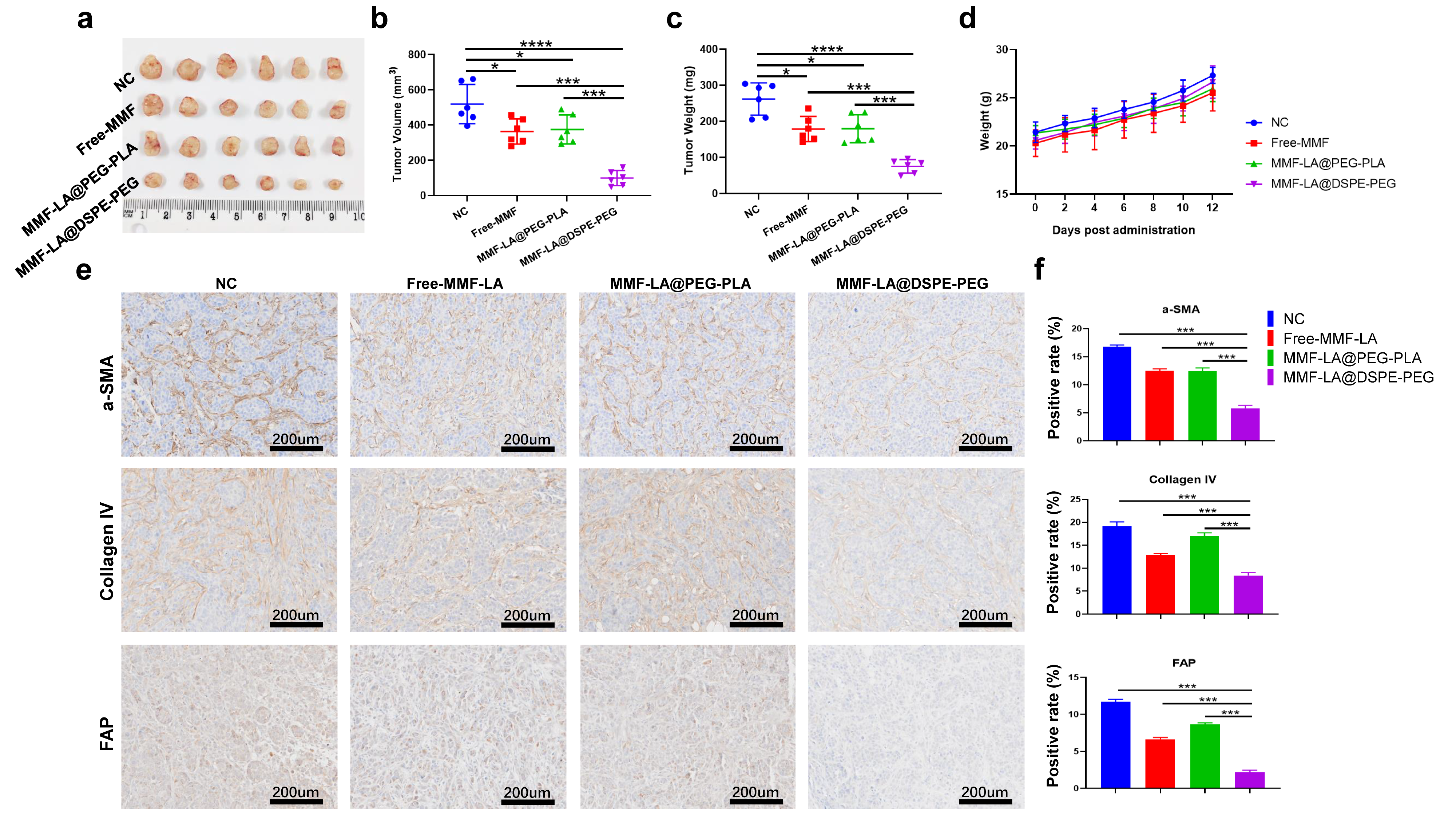

Supplement: Supplementary file 2 — Figure S2 [file JCMM-25-3511-s002.tif]

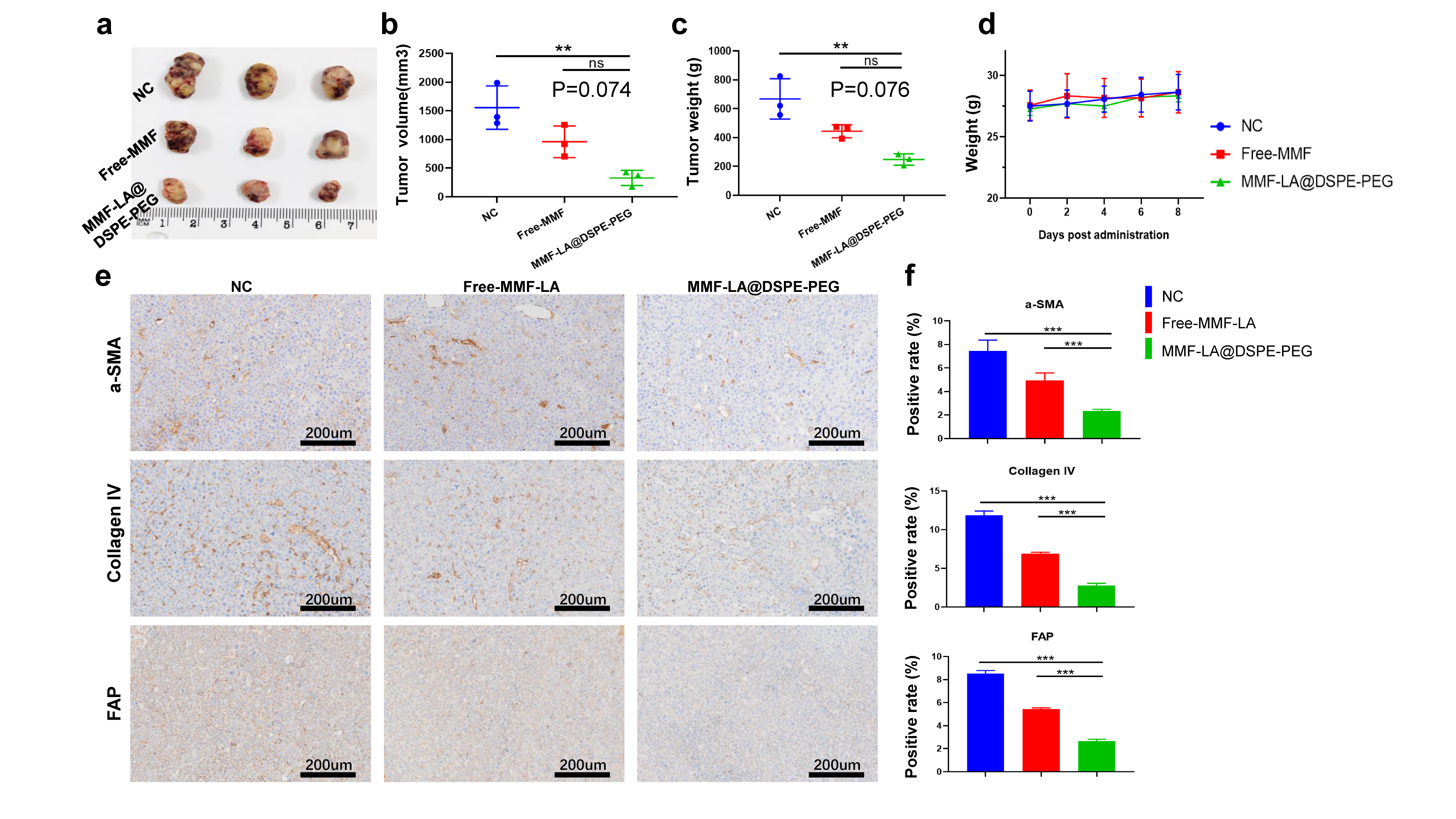

Supplement: Supplementary file 3 — Figure S3 [file JCMM-25-3511-s006.tif]
