# Supplementary material for: Nanoparticle formulation of mycophenolate mofetil achieves enhanced efficacy against hepatocellular carcinoma by targeting tumour‐associated fibroblast
Source: J Cell Mol Med. 2021 Mar 13;25(7):3511–23. doi: 10.1111/jcmm.16434 (PMC8034467; doi:10.1111/jcmm.16434)
Supplement: Supplementary file 4 — Table S1 [file JCMM-25-3511-s007.docx]

**Supplementary Table 1. Clinicopathological Profiles of the Patients**

| Characteristics Number of patients  (Total=68) | |
| --- | --- |
| Age (years), mean ± SD | 52.5 ± 9.6 |
| Sex, n (%) |  |
| Female | 3 (4.41) |
| Male | 65 (95.59) |
| HBV, n (%) |  |
| Yes | 67 (98.53) |
| No | 1 (1.47) |
| †CAF density, n (%) | |
| 1 | 23 (33.82) |
| 2 | 9 (13.24) |
| 3 | 36 (52.94) |
| Microvascular invasion, n (%) | |
| Yes | 16 (23.53) |
| No | 52 (76.47) |
| AFP level, median ng/ml(range) | 46.25 (1.4-62237) |
| Tumor number <=3, n (%) | |
| Yes | 47 (69.12) |
| No | 21 (30.88) |
| Tumor size > 5cm, n (%) | |
| Yes | 28 (41.18) |
| no | 40 (58.82) |
| Pathological grading, n (%) | |
| well | 4 (5.88) |
| median | 33 (48.53) |
| poor | 31 (45.59) |

†CAF: caner associated fibroblast.

Normally distributed continuous variables are presented as mean ± standard deviation (SD), non-normally distributed continuous variables are presented as median (range).
