# Supplementary material for: Nanoparticle formulation of mycophenolate mofetil achieves enhanced efficacy against hepatocellular carcinoma by targeting tumour‐associated fibroblast
Source: J Cell Mol Med. 2021 Mar 13;25(7):3511–23. doi: 10.1111/jcmm.16434 (PMC8034467; doi:10.1111/jcmm.16434)
Supplement: Supplementary file 5 — Table S2 [file JCMM-25-3511-s001.docx]

**Supplementary Table 2. Clinicopathological Characteristics according to** †**CAF Density Score**

| Characteristics | 1+2 (n=32) | 3 (n=36) | P value |
| --- | --- | --- | --- |
| Age (years), mean ± SD | 53.9 ± 8.4 | 51.2 ± 10.4 | 0.236 |
| Gender, n (%) |  |  |  |
| Female | 1 (3.13) | 2 (5.56) | 0.626 |
| Male | 31 (96.87) | 34 (94.44) |  |
| HBV, n (%) |  |  |  |
| Yes | 31 (96.87) | 36 (100) | 0.285 |
| No | 1 (3.13) | 0 (0) |  |
| Microvascular invasion, n (%) | | |  |
| Yes | 3 (9.38) | 13 (36.11） | 0.009 |
| No | 29 (90.62) | 23 (63.89) |  |
| AFP level, median- ng/ml(range) | 26.2 (1.5-23492) | 65.9 (1.4-62237) | 0.125 |
| Tumor number <=3, n (%) | | |  |
| Yes | 23 (71.88) | 24 (66.67) | 0.643 |
| No | 9 (28.12) | 12 (33.33) |  |
| Tumor size > 5cm, n (%) | | | 0.283 |
| Yes | 11 (34.38) | 17 (47.22) |  |
| no | 21 (65.63) | 19 (52.78) |  |
| Pathological grading, n (%) | | | 0.089 |
| well | 4 (12.50) | 0 (0) |  |
| median | 14 (43.75) | 19 (52.78) |  |
| poor | 14 (43.75) | 17 (47.22) |  |

†CAF: caner associated fibroblast.

Normally distributed continuous variables are presented as mean ± standard deviation (SD), non-normally distributed continuous variables are presented as median (range).
