# Supplementary material for: Nanoparticle formulation of mycophenolate mofetil achieves enhanced efficacy against hepatocellular carcinoma by targeting tumour‐associated fibroblast
Source: J Cell Mol Med. 2021 Mar 13;25(7):3511–23. doi: 10.1111/jcmm.16434 (PMC8034467; doi:10.1111/jcmm.16434)
Supplement: Supplementary file 6 — Method S1 [file JCMM-25-3511-s004.docx]

**Supplementary Methods**

**Colony formation assay**

Cells were trypsinized and suspended into single-cell at a density of 1 × 10^3^ cells/ml in DMEM. 2000 cells were seeded in each well of 6-well plates and allowed to grow for 96 h. Then, cells were cultured in medium with or without Drugs for another 96h. Cell colonies were fixed by 4% formaldehyde and were further stained by crystal violet.

**Western blot analysis**

Protein concentrations were determined by BCA assay kit (Thermo, America). Cell lysates were separated by SDS-PAGE electrophoresis and transferred onto polyvinylidene fluoride (PVDF) membranes. Then, the membranes were blocked by 5% fat-free milk for 1h and incubated with primary antibodies at 4°C for one night. Primary antibodies used in this study were listed as follows: rabbit anti-cyclin D (1:1000) (CST, America), rabbit anti-cyclin E (1:1000) (CST, America), rabbit anti-tubulin (1:1000) (CST, America), rabbit anti-GAPDH (1:1000) (CST, America). After incubation, these membranes were washed for three times and reacted with horseradish peroxidase-conjugated secondary antibody for 1h. Proteins on the membrane were visualized using ECL Prime Western Blotting Detection Reagent (Thermo, America).

**Immunohistochemistry (IHC)**

Primary antibodies used in this study were listed as follows: rabbit anti-PCNA (1:1000) (Abcam, UK), rabbit anti-α-SMA (1:500) (CST, America), rabbit anti-FAP (1:200) (Abcam, UK), rabbit anti-collagen IV (1:200) (Abcam, UK), rabbit anti-CD31 (1:50) (Abcam, UK). The expression levels of these genes were quantified using Image J 1.48u software by calculating the relative rate of positive staining area. The CAF density was scored as 1: relative rate of positive staining area < 3%; 2: 3% ≤ relative rate of positive staining area < 6%; 3: relative rate of positive staining area ≥ 6%.

**Boyden chamber migration assay**

Cells resuspended in 200ul FBS-free medium were seeded in the upper chamber of 24-well Transwell plate with a density of 5× 10^4^ cells/well, and the bottom chamber was added with 1ml complete medium (10%FBS). Cells were treated with MMF (2~~ug~~ µg/ml) or MMF-LA NPs (at 2~~ug~~ µg /ml MMF-equivalent dose). After 24h incubation, cells remained in the upper chamber were removed and the migrated cells were stained using 0.5% crystal violet.
