# Supplementary material for: Nanoparticle formulation of mycophenolate mofetil achieves enhanced efficacy against hepatocellular carcinoma by targeting tumour‐associated fibroblast
Source: J Cell Mol Med. 2021 Mar 13;25(7):3511–23. doi: 10.1111/jcmm.16434 (PMC8034467; doi:10.1111/jcmm.16434)
Supplement: Supplementary file 7 — FigCaption [file JCMM-25-3511-s003.docx]

**Figure S1** (a) Expression levels of α-SMA and Tubulin in LX2 cells determined by Immunofluorescence. The scale bars: 50 um. (b) Cell viability of LX2 cells treated with free MMF, MMF-LA@DSPE-PEG and MMF-LA@PEG-PLA for 48h. (c) LX2 cells were seeded in the top chamber of transwell with serum-free medium and treated with free MMF, MMF-LA@DSPE-PEG and MMF-LA@PEG-PLA. After 24 h, migrated cells were fixed, stained and photographed. The scale bars: 200 um (d) Quantitative analysis of the migrated cell number, data are shown as the mean ± SD, (n = 3), ***P < 0.001

**Figure S2** MMF-LA@DSPE-PEG inhibited HCC growth and reduced CAF density in highly fibrotic HCC Model II. Mice were orally administrated with free MMF (20mg/kg) or intravenously injected with MMF-LA NPs (at 20mg/kg MMF-equivalent dose) every other day for five times. (a) Tumour images of different groups, (n=6). (b) Tumour volume of different groups, (mean ± SD, n=6), *P < 0.05, ***P < 0.001, ****P < 0.0001. (c) Tumour weights of different groups, (mean ± SD, n=6), *P < 0.05, ***P < 0.001, ****P < 0.0001. (d) Bodyweights of mice in different groups, (mean ± SD, n=6). (e) Expression levels of α-SMA, collagen IV and FAP were determined by Immunohistochemistry. The scale bars: 200 um. (f) Quantitative analysis of panel E (Image J software), data are shown as the mean ± SD, (n = 3), ***P < 0.001.

**Figure S3** MMF-LA@DSPE-PEG inhibited HCC growth and reduced CAF density in PDX models. Mice were orally administrated with free MMF (20mg/kg) or intravenously injected with MMF-LA NPs (at 20mg/kg MMF-equivalent dose) every other day for five times (a) Tumour images of different groups, (n=3). (b) Tumour volume of different groups, (mean ± SD, n=3), **P < 0.01. (c) Tumour weights of different groups (mean ± SD, n=3), **P < 0.01. (d) Bodyweights of mice in different groups, (mean ± SD, n=3). (e) Expression levels of α-SMA, collagen IV and FAP were determined by Immunohistochemistry. The scale bars: 200 um. (f) Quantitative analysis of panel E (Image J software), data are shown as the mean ± SD, (n = 3), ***P < 0.001.
